# Differential Accumulation of Innate- and Adaptive-Immune-Response-Derived Transcripts during Antagonism between Papaya Ringspot Virus and Papaya Mosaic Virus

**DOI:** 10.3390/v12020230

**Published:** 2020-02-19

**Authors:** Pablo Vargas-Mejía, Julio Vega-Arreguín, Gabriela Chávez-Calvillo, Enrique Ibarra-Laclette, Laura Silva-Rosales

**Affiliations:** 1 Laboratorio de Ciencias AgroGenómicas, Escuela Nacional de Estudios Superiores, Universidad Nacional Autónoma de México, León, Gto. 37684, Mexico; pablovargasmejia@gmail.com (P.V.-M.); jvega.arreguin@gmail.com (J.V.-A.); 2 Departamento de Ingeniería Genética, Centro de Investigaciones y de Estudios Avanzados, Unidad Irapuato Libramiento Norte Carretera Irapuato-León, Irapuato, Gto. 36824, Mexico; gabvet.c@gmail.com; 3 Red de Estudios Moleculares Avanzados, Instituto de Ecología A.C. Carretera Antigua a Coatepec #351, Xalapa, Ver. 91070, Mexico; ibarralaclette206@gmail.com; 4 Biological Sciences, 121 Rouse Life Sciences Building, Auburn University, Auburn, AL 36849, USA

**Keywords:** antagonism, cross protection, PRSV, PapMV, host resistance, plant–virus interaction, potyvirus, superinfection exclusion, papaya genomics

## Abstract

Papaya ringspot virus (PRSV), a common potyvirus infecting papaya plants worldwide, can lead to either antagonism or synergism in mixed infections with Papaya mosaic virus (PapMV), a potexvirus. These two unrelated viruses produce antagonism or synergism depending on their order of infection in the plant. When PRSV is inoculated first or at the same time as PapMV, the viral interaction is synergistic. However, an antagonistic response is observed when PapMV is inoculated before PRSV. In the antagonistic condition, PRSV is deterred from the plant and its drastic effects are overcome. Here, we examine differences in gene expression by high-throughput RNA sequencing, focused on immune system pathways. We present the transcriptomic expression of single and mixed inoculations of PRSV and PapMV leading to synergism and antagonism. Upregulation of dominant and hormone-mediated resistance transcripts suggests that the innate immune system participates in synergism. In antagonism, in addition to innate immunity, upregulation of RNA interference-mediated resistance transcripts suggests that adaptive immunity is involved.

## 1. Introduction

Despite the acceptance of terms coined for the plant–fungal and plant–bacterial models, which were adapted for viruses, the immune system in plants as a response to viral infections still lacks a uniform general descriptive framework [1,2]. For viruses, two types of immunity are described: innate and adaptive. For the first, three types of resistance mechanisms were reported: (1) dominant resistance, which is called “gene-for-gene”, mediated by canonical dominant resistance, R proteins of the type nucleotide-binding sequence leucine-rich repeat (NBS-LRR), and characterized by the molecular pathway of plant–pathogen interactions leading to hypersensitive responses (HRs) [3,4]; (2) recessive resistance, usually achieved by the incompatible interaction between viral proteins and host factors, such as eIF4E and eIF4G (eukaryotic translation initiation factors 4E and 4G respectively) [5,6]; and (3) hormone-mediated resistance, such as ethylene, salicylic acid, and jasmonic acid, triggering resistance through their recognition by specific receptors [7,8,9]. The plant adaptive immune system is often RNA interference (RNAi)-mediated resistance that occurs after an elapsed time of infection. In this case, double-stranded RNA (dsRNA) is considered the microbe- or pathogen-associated molecular pattern (MAMP or PAMP, respectively) [2], thus leading to an antiviral silencing mechanism [10,11]. This can be observed in the oxidative burst triggered by dsRNA of Oilseed rape mosaic virus (ORMV) and Plum pox virus (PPV) in *Arabidopsis* and *Nicotiana* species, respectively [12,13,14]. RNAi is a well-conserved defense mechanism against viruses in eukaryotes, mediated by dicer-like enzymes (DCL), Argonaute (AGO) proteins, and RNA-dependent polymerases (RDR) [15,16].

As viruses are the most abundant organisms in the natural environment, mixed infections are common [17,18,19,20,21,22], and they can result in three types of virus–virus interactions: (1) neutralism, when the presence of one virus does not affect the other; (2) synergism, when one or both viruses facilitate the other in replication, translation, movement, or transmission; and (3) antagonism, when one or both viruses hinders replication, translation, movement, or transmission of the other [23,24,25]. These types of complex interactions raise questions about the involvement of the different components of the immune responses in the plants. In this field, studies mainly focused on single infections [26] and only recently on mixed infections [27,28,29].

The system in our study involved infection with two viruses: Papaya ringspot virus (PRSV), a positive single-strand (+ss) RNA member of the *Potyviridae* family with a genome of 10.33 kb and distributed worldwide, causing crop losses of 10% to 100% [30]; and Papaya mosaic virus (PapMV), an *Alphaflexiviridae* family member with a +ssRNA of a 6.66-kb genome.

Previously, our group reported the first antagonistic interaction between these two non-related viruses [31]. Their mixed infection develops either synergistic or antagonistic interactions, depending on the order of infection in papaya plants. When PRSV infects first or co-infects with PapMV, the viral interaction becomes synergistic. However, an antagonistic response is observed when PapMV infects first, followed by PRSV. In the same study, we provided biochemical and molecular evidence regarding the ability of PRSV to make better use of plant translational machinery compared with PapMV. PapMV infection was associated with a higher expression of two biochemical landmarks of the systemic acquired resistance (SAR): pathogenesis-related protein 1 (PR1), and reactive oxygen species (ROS) [31]. Both responses, higher in antagonism than in synergism or PRSV infection, reflect immune activity in the plant.

Here, we give evidence of how the activation of different immune mechanisms occurs, comparing the single infections of PRSV and PapMV, and co-infections that produce synergism (PapMV + PRSV) and antagonism (PapMV→PRSV). Our analysis contributes to the understanding of the underlying gene activation of innate or adaptive plant immunity responses that express differentially during synergism and antagonism by high-throughput RNA sequencing (RNA-Seq). We found that single infection of PRSV triggers components of innate immunity (dominant and hormone-mediated resistance); PapMV involves both adaptive and innate immunity (RNAi and dominant resistance). Antagonism (PapMV→PRSV) involves both as well, with a higher number of upregulated genes of dominant and hormone- and RNAi-mediated resistances. Unexpectedly, the gene expression profiles during synergism (PapMV + PRSV) and antagonism (PapMV→PRSV) were similar. After functional enrichment analysis, the condition of synergism (PapMV + PRSV) was the most dissimilar to antagonism (PapMV→PRSV) and did not upregulate immunity-related genes.

Since both single PRSV and PapMV infections can trigger components of the innate system (dominant and hormone-mediated resistance), we hypothesized that, during antagonism (PapMV→PRSV), PRSV is counteracted by the PapMV initial immune response through the onset of adaptive immunity (RNAi).

## 2. Materials and Methods

### 2.1. Virus and Plant Materials

We performed single (PapMV or PRSV), simultaneous (PapMV + PRSV), and stepwise (PapMV→PRSV, PRSV→PapMV) inoculations in *Carica papaya* plants. As controls, we used mock-inoculated plants. Plants of *C. papaya* var. Maradol were cultivated in an insect-free greenhouse in summer or fall. Seeds were germinated in a mix containing 1:1 coconut paste and growing substrate Sun Shine Mix 3 (Agawan, MA, USA). When cotyledons emerged, seedlings were transplanted to commercial growing substrate. Plants with five true leaves (approximately six weeks old) were used for all experiments. The plants were inoculated with dusted carborundum (400 mesh) and 5 μL of viral solution composed of powder tissue from infected plants (12 ng of PRSV tissue and 7.6 ng of PapMV tissue), 1 mM sodium phosphate buffer (pH 8.0), and 1 mM ethylenediaminetetraacetic acid (EDTA). The estimated virus amounts were calculated as described previously [31]. Mock-inoculated plants were dusted with carborundum and treated with buffer alone. The initial inoculation was conducted on the third leaf of each plant with sterile cotton buds soaked in the viral solution and scraping the basal part of the leaf. The second inoculation was performed 30 days after the first inoculation. The viruses used here were PRSV from the state of Colima (AF309968), Mexico, and PapMV from Guanajuato, Mexico (PapMV-Gto), collected as described previously [29,30]. To confirm both virus isolates identities, we used an enzyme-linked immunosorbent assay (ELISA) with commercial Agdia, (Elkhart, IN, USA)) antibodies against the coat protein (CP) of each virus (α-PapMV 53400 and α-PRSV 53500) and RT-PCR to amplify and sequence the CP of both viruses as described previously [31].

### 2.2. Experimental Design

Two experimental replicates were used, each containing 48 papaya plants grown in an insect-free greenhouse. In each experiment, papayas were divided into six groups of eight plants each, separated per treatment. Treatments were divided as one-inoculation and two-inoculation groups. One-inoculation treatment consisted of plants inoculated once at time zero, with PapMV, PRSV, mock, or PapMV + PRSV simultaneous inoculation. The two-step treatments consisted of a first inoculation with PRSV at time zero, and at 30 days post infection (dpi) with PapMV, referred to as PRSV→PapMV, or PapMV→PRSV, consisting of a first inoculation with PapMV, at time zero, and then with PRSV at 30 dpi. The second inoculation was performed on the eighth leaf when plants had about 13 to 15 leaves. Samples for RNA extraction were collected from systemic leaves at 60 dpi, when all the infection phenotypes were fully established. Damage was evaluated as previously described [31].

### 2.3. Illumina Sequencing

Total RNA was extracted from 100 mg of frozen tissue taken from 400 mg of pooled tissue derived from the first systemic leaves above the inoculated leaf (ninth leaf) from four plants and ground in liquid nitrogen. RNA isolation was performed using TRIzol reagent (ThermoFisher, Waltham, MA, USA), followed by a DNAseI (Thermo Fisher Scientific, Waltham, MA, USA) treatment according to the manufacturer’s instructions. A total of 12 paired-end (2 × 100) RNA-Seq libraries (six treatments, two replicates per treatment) were prepared and sequenced at Cinvestav facilities with Illumina HiSeq2500 (Hayward, CA, USA). Raw data are publicly available at the National Center for Biotechnology Information (NCBI), BioProject accession PRJNA560275.

### 2.4. De Novo Assembly, Mapping, and Statistical Analysis

Trimming adaptors and cleaning of duplicated and low-quality reads were conducted as described before [32]. For the de novo assembly, paired-end reads of all conditions were merged and normalized with the Trinity “insilico_read_normalization.pl” script. Then, the resultant merged reads were used for de novo assembly with Trinity, under standard options [33]. To map the reads, quantify transcripts, and obtain gene abundances, we used Kallisto [34] as the estimation method. Kallisto was run with bias correction and a bootstrap number of 1000. Differential expression analysis was conducted with Sleuth performed as reported before [35]. Transcripts with log2 fold change (log2FC) values ≥1.5 or ≤−1.5 and *q*-values ≤0.05 were declared as differentially expressed genes (DEGs).

### 2.5. Functional Annotation

The coding sequence (CDS) of each gene and transcript was annotated using TransDecoder [33]. Differentially expressed genes (DEGs) were annotated based on top-BLASTx-hit similarity searches against The *Arabidopsis* Information Resource (TAIR 10) and SwissProt databases under a threshold e-value ≤ 1 × 10^5^. Each DEG was functionally classified and functionally annotated based on eggNOG with a Gene Ontology (GO) classification source in terms of their biological processes (BP), molecular function (MF), and cellular component (CC) using the AgriGO analysis tool [36,37].

To identify enrichment of GO terms and differences between treatments, a cross-comparison of singular enrichment analysis (SEA) was performed with the AgriGO v2 tool under the multi-test adjustment method of Hochberg (FDR) with a *p*-value cut-off of 0.05 [37]. Reduction of ontology and construction of the network were performed with REVIGO and Cytoscape [38]. For this analysis, we used DEGs showing high homology to TAIR 10 genes. GeneCodis was used to perform the enrichment analysis of Kyoto Encyclopedia of Genes and Genomes (KEGG) pathways and modular enrichment analysis with a hypergeometric statistical test under a *p*-value cut-off of 0.05 [39].

### 2.6. Gene Expression Validation

Bioinformatic results of gene expression were validated using quantitative RT-PCR, using the same source of RNA as for the RNA-Seq samples. Complementary DNA (cDNA) was synthesized using 2 µg of total RNA with RevertAid H minus enzyme according to the manufacturer’s instructions (Thermo Scientific, Waltham, MA, USA). RT-PCR reactions for 12 selected genes (Table S1, Supplementary Materials) were performed in an CFX96 Real time system (BioRad, Hercules, CA, USA) with the NZY qPCR Green Master Mix (2×) (NZYTech, Lisbon, Portugal) according to the manufacturer’s instructions. Relative expression was performed using the Livak method (2^−ΔΔCq^) with β-tubulin as the housekeeping gene. The oligonucleotides for the β-tubulin gene were 458-β-tub-F AGTGATTTTCCCGGGTCAGCTCAA (forward) and 459-β-tub-R TGCTGCCTGAGGTTCCCTGGT (reverse).

## 3. Results

### 3.1. Symptom Development with Single and Mixed Infections of PapMV and PRSV

**Figure 1:**
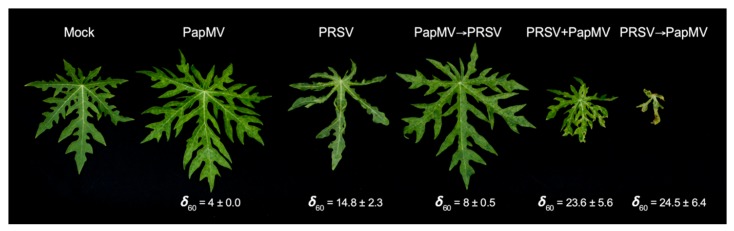
Leaves of mock-inoculated and virus-infected plants showing symptoms at 60 days post infection (dpi) with damage values (δ_60_). Single infections of PapMV (Papaya mosaic virus), PRSV (Papaya ringspot virus), stepwise infection of PapMV→PRSV resulting in antagonism, stepwise infection of PRSV→PapMV, and co-inoculation of PRSV with PapMV, resulting in synergism.

We previously reported that plants infected with PapMV showed systemic disease symptoms in less time (at about 5 dpi), which were less severe than those infected with PRSV, whose symptoms were evident at about 19 dpi [31]. PRSV infections produced severe deformation of the leaves with foliar mass reduction, mosaics, chlorosis, and vein yellowing [31]. PapMV infections resulted in mild mosaics, like those observed in the sequential infections, which led to viral antagonism (PapMV→PRSV), as reported before, with a damage value at 60 dpi (δ_60_) significantly greater than the PapMV single infection (PapMV→PRSV δ_60_ = 8 ± 0.5 and PapMV δ_60_ = 4 ± 0.0). The damage caused by this type of infection was less severe than by PRSV alone (δ_60_ = 14.8 ± 2.3). Mixed infections leading to synergisms with stepwise PRSV→PapMV and simultaneous PapMV + PRSV inoculations showed the most plant damage (PRSV→PapMV δ_60_ = 24.5 ± 6.4 and PapMV + PRSV δ_60_ = 23.6 ± 5.6). Symptoms resulting from the two types of synergistic conditions (PRSV→PapMV and PapMV + PRSV) included systemic necrosis, partial defoliation, apical necrosis, plant stunting, leaf mosaics, and leaf deformation (Figure 1). These responses were consistent along the years in our greenhouses. Similar phenotypes were reported for other potyvirus–potexvirus mixed infections [40]. Single and mixed infections of PapMV and PRSV occur in Mexican papaya crops in the field [41]. These results indicate that a complex interaction between both viruses and the host plant occur that depend on the first infecting virus, triggering different responses of the plant.

### 3.2. RNA-Seq of Virus-Infected Papaya Plants from Single and Mixed Infections

To explore and analyze the global host plant response to single and mixed viral infections with PapMV and PRSV, we obtained transcriptomes by RNA-Seq of plants infected with PapMV, PRSV, PapMV→PRSV, PapMV + PRSV, and PRSV→PapMV. Mock inoculated plants were also included. The number of reads per library is available in Table S2 (Supplementary Materials). The assembled transcriptome of the six conditions generated 149,288 transcripts corresponding to 63,243 unigenes with an average length of 1307 bases. To avoid redundancy, we chose only one transcript per unigene based on the top-most highly expressed transcripts and the longest isoform per unigene. The mean of pseudo aligned reads with Kallisto was 92.53%, of which 85.41% had only one pseudo alignment to the transcriptome (Table S1, Supplementary Materials). To corroborate the quality of the experimental replicated libraries, we clustered the transcripts per million (TPM) and estimated counts of each sample using the FlashClust library with the hclust plot and an average method. Due to the lack of repeatability of the PRSV→PapMV replicates (Figure S1, Supplementary Materials), we decided to not consider this condition of synergism in further analyses.

### 3.3. Gene Expression Profiling and Functional Annotation Addresses Antagonism Highly Enriched in Immune-Related Response

**Figure 2:**
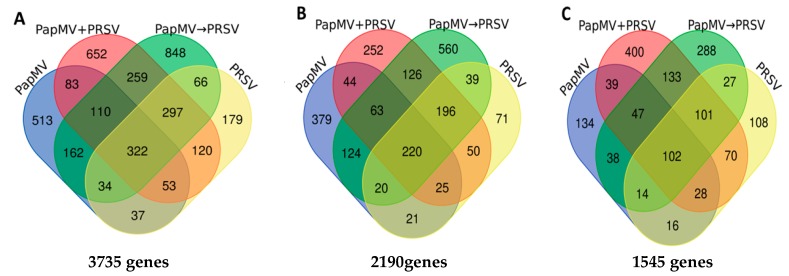
Differentially expressed genes (DEGs) on a Venn diagram for the single infections of PapMV and PRSV, and the stepwise infections of PapMV→PRSV (antagonism) and PapMV + PRSV (synergism). PapMV→PRSV (green), PapMV + PRSV (red), PapMV (blue), and PRSV (yellow). (**A**) Total DEGs (up- and downregulated). (**B**) Upregulated genes with ≥1.5 b (foldchange-like) values. (**C**) Downregulated genes with ≤−1.5 b (foldchange-like) values.

Genes with log_2_ fold change (FC) values ≥ 1.5 or ≤ −1.5 and *q*-values ≤ 0.05 were considered to be DEGs. A total of 3735 genes were differentially expressed in the plant in response to the four viral infected conditions (Figure 2). For all conditions, a higher number of upregulated (2190) than downregulated (1545) DEGs was found (Figure 2). For the antagonistic condition (PapMV→PRSV), 1.8-fold more genes (1348) were upregulated compared to those downregulated (750). Similar contrasting numbers were found for the single PapMV infection with 896 upregulated and 418 downregulated genes. Fewer differences were found in the PRSV condition with 642 up- and 466 downregulated genes. Similar numbers of 976 and 920 for the up- and downregulated genes, respectively, were observed in the synergistic PapMV + PRSV condition (Figure 2). PapMV→PRSV (antagonism) and PapMV + PRSV (synergism) shared more DEGs with each other (605 upregulated and 383 downregulated) than with any single infection.

Notably, for antagonism (PapMV→PRSV), more upregulated DEGs were shared with PRSV (39 + 196 + 220 + 20 = 475) than with PapMV single infections (63 + 124 + 20 + 220 = 427), with a difference of 48, indicating that PapMV single infection is the most dissimilar condition in terms of the number of differentially expressed genes to antagonism (PapMV→PRSV).

**Figure 3:**
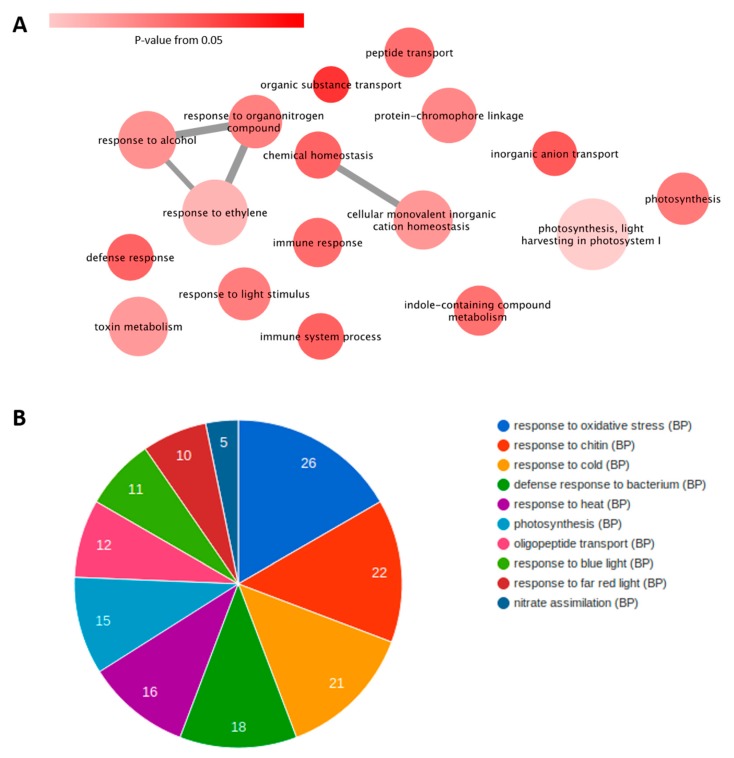
Biological processes (BPs) and their functional annotation in antagonism (PapMV→PRSV). (**A**) Network of non-redundant unique BPs in antagonism. Edges represent relationships between the BPs (nodes). Node color intensity denotes the *p*-value of the sample. Size of the node represents the number of DEGs. (**B**) Number of DEGs for each BP, per concurrent annotations, through a modular enrichment analysis for PapMV→PRSV.

To understand the molecular aspects of the antagonism (PapMV→PRSV) and their relationship with the other conditions, we used three comparative strategies. The first one consisted of a singular enrichment analysis (SEA) for each condition, followed by a reduction in redundant ontologies of biological processes (BP) with REVIGO and then the generation of an ontology network. The networks from the four conditions, single PRSV and PapMV infections, PapMV→PRSV (antagonism), and PapMV + PRSV (synergism), were collapsed into one network to only show the differences in antagonism (PapMV→PRSV) against all the other conditions (Figure 3A). Individual networks are available in Figure S2 (Supplementary Materials). This final network shows the most important biological process (BP) in antagonism (PapMV→PRSV). The most notable BPs were response to ethylene, toxin metabolism, defense response, immune response, response to light stimulus, light harvesting in photosystem I, and photosynthesis. Some of the genes involved in these BPs are listed in Table S3 (Supplementary Materials). A strong response to light stimulus, photosynthesis, and light harvesting in photosystem I was found. Next, using a modular enrichment analysis (Figure 3B), we found several responses to light, such as response to red, blue, and far-red lights, and others such as oxidative stress and chitin, which were not identified in the network approach.

**Figure 4:**
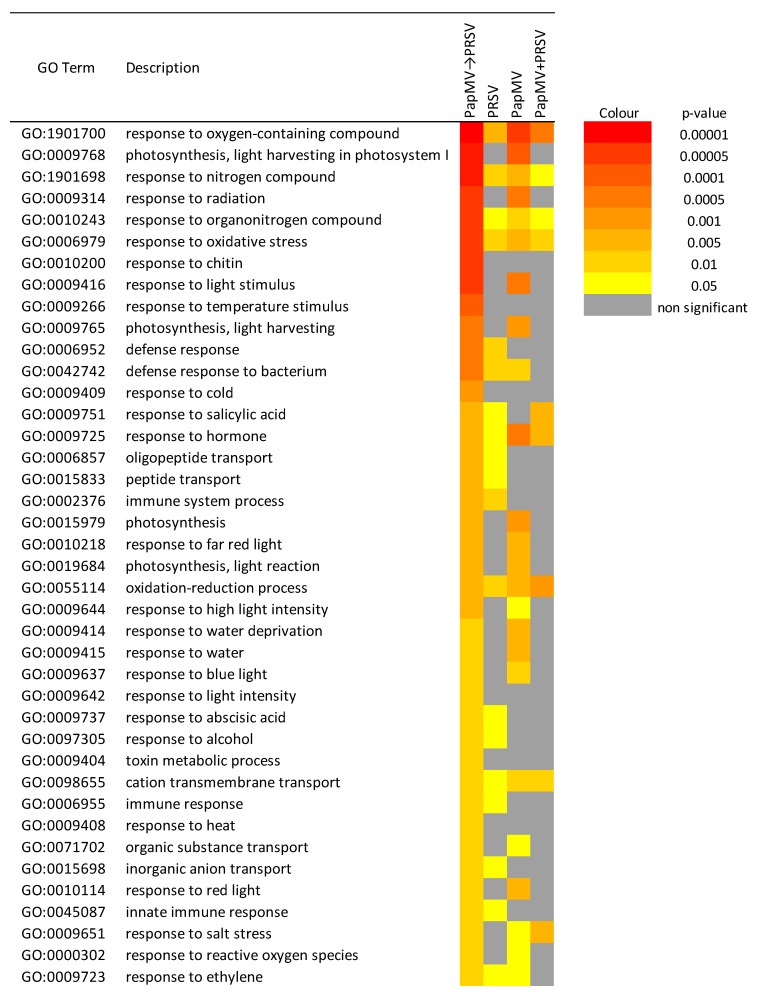
Color map of the cross-comparison of single enrichment analysis (SEACOMPARE). Color scale represents the significance level of ontology (BPs), for each condition. The figure only depicts those Gene Ontology (GO) terms that met the criteria of low redundancy OR present in the network unique for antagonism (PapMV→PRSV) OR shared with other conditions AND significant for PapMV→PRSV.

The next approach consisted of two types of cross comparisons of SEA (SEACOMPARE) for each condition with upregulated and downregulated DEGs. From this analysis, we chose GO terms that met the criteria: low redundant ontologies OR present in the network unique for PapMV→PRSV OR shared with other conditions AND significant for PapMV→PRSV (Figure 4). We found similar results between SEACOMPARE and the network analysis, but SEACOMPARE provided more complete insight. Firstly, the abovementioned responses to chitin, light, photosynthesis, light harvesting in photosystem I, oligopeptide transport, and responses concerning oxygen species (oxidative stress) appeared again, but immune and defense responses were also detected. Secondly, new enrichments appeared, such as responses to temperature stimulus, cold, heat, salt stress, and water, associated with abiotic stress. Five biological processes were found only in antagonism (PapMV→PRSV): responses to chitin, temperature stimulus, cold, heat, light intensity, and toxin metabolic process. General defense/stress processes (defense response, response to hormone, etc.) and specific processes (response to chitin, immune system, cold, etc.) that were shared for a different set of conditions are depicted in Figure 4. Defense and immune responses are enriched in antagonism and PRSV infection, whereas the defense response to bacteria is enriched in antagonism and PapMV infection. These findings highlight the contribution of each virus in this condition, where PRSV is attenuated. Notably, no processes are shared only between antagonistic (PapMV→PRSV) and synergistic (PapMV + PRSV) conditions.

**Figure 5:**
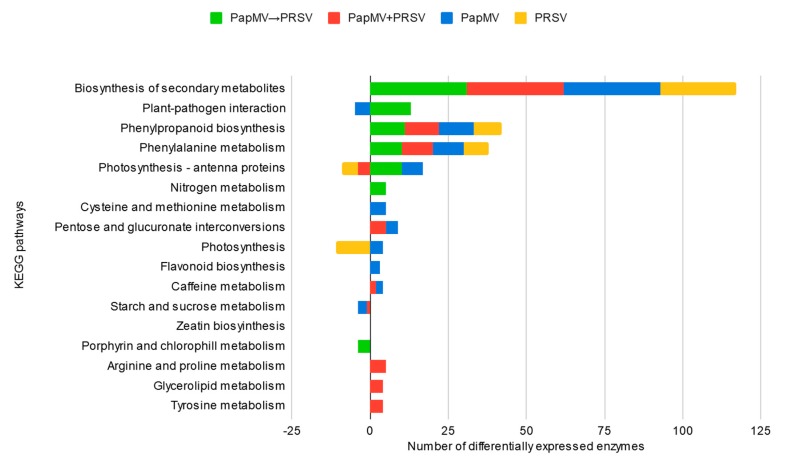
Metabolic pathways involved in antagonism, synergism, and single infections. Stacked bar chart of the enriched KEGG pathways (Kyoto Encyclopedia of Genes and Genomes). The *x*-axis represents the number of differentially expressed enzymes.

The last approach involved an enrichment analysis of reported metabolic pathways in KEGG. For this analysis, we subtracted all the annotated enzymes from the DEGs and mapped their identifications (IDs) to KEGG (Figure 5). Out of 16 metabolic pathways enriched in all conditions, we found upregulated enzymes for biosynthesis of secondary metabolites, phenylpropanoid biosynthesis, and phenylalanine metabolism. Other pathways are differentially enriched. Plant–pathogen interaction is enriched in both antagonism (with upregulated enzymes) and PapMV (with downregulated enzymes). Due to the phenotype of PRSV infection and synergism, photosynthesis metabolism is enriched with downregulated enzymes in both conditions as expected. In PapMV infection, porphyrin and chlorophyll metabolism include downregulated enzymes. The remaining metabolic pathways have upregulated enzymes for cysteine and methionine, as well as flavonoid biosynthesis for PapMV, and upregulated enzymes for pentose and glucuronate interconversions and caffeine metabolism for both PapMV and synergism. For the same two conditions, starch and sucrose metabolism are downregulated. Three metabolic pathways (arginine and proline, glycerolipid, and tyrosine metabolisms with upregulated enzymes) were only found for synergism. Finally, nitrogen metabolism with upregulated enzymes is only enriched in antagonism and corresponds to the organonitrogen compound response seen in Figure 3A. Validation of bioinformatic results via qRT-PCR was carried out for the following 12 candidate genes: *WRKY 18*, *WRKY 33*, *WRKY 53*, *TIR-NBS-LRR*, *RDR1*, *DCL2*, *DCL4*, *AGO2*, *RBOHD*, *SOD1*, *LOX2*, and *LRR-RK*. For all genes, except *RBOHD*, real-time quantification corroborated the accuracy of bioinformatic analyses (Table S1, Supplementary Materials).

## 4. Discussion

Mixed infections in papaya plants were documented for some years and more recently in Mexico with up to 15 viruses [21]. However, the mechanisms that underlay the production of symptoms under different viral combinations of mixed infections require further analysis. We previously provided insight into the antagonistic response triggered by the sequential infection of a potex (PapMV) and a potyvirus (PRSV), which allowed us to gather information to confirm the ability of PRSV to efficiently divert the plant translational machinery to favor its own genome translation [31]. We also provide evidence of ROS species and a PR1 marker protein for SAR, being highly expressed in PapMV infection. We unveiled the participation of different components of the innate and adaptive immune systems through a transcriptomic analysis comparing the conditions of antagonism, synergism, and single infections to understand how PRSV, a virus that efficiently hijacks the translation machinery of the plant and has a counter silencing protein (HC-Pro) [1], succumbs to PapMV.

### 4.1. Potyvirus PRSV Triggers Innate Immunity and Potexvirus PapMV Triggers Adaptive Immunity

PRSV triggers the plant defense and immune system as reported with other potyviral infections, like the adapted TEV-*At*17b strain to L*er*-0 *Arabidopsis thaliana* (tested with seven ecotypes Col-0, Di-2, Ei-2, L*er*-0, Oy-0, St-0, and Wt-1) and the infection of soybean with Soybean mosaic virus (SMV), which triggers the response to stimulus and the signaling pathways of salicylic acid, jasmonic acid, and ethylene [42,43]. Our enrichment analyses showed biological responses of light, blue light, chitin, and innate immune responses, similar to those found in susceptible and resistant cassava varieties infected with the ipomovirus Cassava brown streak virus (CBSV) [44] and with susceptible and resistant apricot cultivars to Plum pox virus (PPV) [45]. Even though members of the *Potyviridae* family can trigger genes labeled with biological processes like defense responses, innate immune response, immune system process, and response to chitin in the cassava susceptible to CBSV, or abiotic stimulus response in the PPV in susceptible apricot cultivars, these do not seem to be enough to generate resistance in plants, and resistant cultivars do not owe their resistance to the sole expression of defense genes involved in these processes [44,45]. Immune responses through transcriptome analyses were reported for the synergist infection of panicovirus Panicum mosaic virus (PMV) and its satellite (SPMV) infecting the monocot *Brachypodium distachyon* through the expression of pathogenesis related proteins 1, 3, and 5 and proteins with WRKYGQK domain transcription factors (*PR1*, *PR3*, *PR5*, and *WRKY53*) [27]. In the case of the antagonistic partnership of PRSV, PapMV adaptive immunity is triggered as the RNA-mediated silencing system is turned on (Table 1), shown by the upregulation of genes for dicer-like ribonucleases 2–4, RNA-dependent RNA polymerases, and Argonaute 2 (*DCL2–4*, *RDR1*, and *AGO2*), as well as responses to reactive oxygen species (ROS), such as superoxide dismutase 1, lipoxygenase 2 and respiratory burst oxidase homolog protein D (*SOD1*, *LOX2*, and *RBOHD*). In other plants such as *Arabidopsis* sp. or *Benthamiana* sp. infected with PVX, the RNAi machinery was suggested to limit potexvirus virulence through CP recognition by *NB-LRR* proteins that directly trigger the RNAi system [46,47,48,49]. For papaya antagonism, we speculate that ROS are enhanced due to changes in the photosynthetic responses to blue, far-red, and red light (Figure 3B and Figure 4), highly expressed in PapMV→PRSV and PapMV. Plant defense resistance mechanisms are proposed to tightly cross-communicate with general signaling pathways to enable efficient pathogen recognition [50]. In the case of light responses, pathogen infection reduces photosynthetic activity, as does PRSV, leading to a reprogramming of carbon metabolism and, therefore, to the expression of defense-related genes and chloroplast-derived ROS [51,52]. Infection with PapMV generates a transcriptomic profile similar to that produced by Papaya meleira virus (PMeV) infection in papaya plants, with expression of *PR1*, and ROS-related genes, but with lower expression of other defense-related genes, and no differential expression in the silencing machinery [53]. This would suggest that the antagonistic interaction could occur in combinations of PRSV with other viruses like PMeV, as these mixed infections occur in natural environments [15].

### 4.2. Antagonism and Synergism: Similar Expression, Large Differences

Synergistic and antagonistic interactions are the two outcomes of a mixed infection with PapMV and PRSV; however, they are similar in terms of the responses elicited by each. If we consider the network analysis in Figure S2 (Supplementary Materials), PapMV→PRSV and PapMV + PRSV (antagonism and synergism) are similar in many of the biological process nodes. The only differences between them are the representative nodes and edges in antagonism shown in Figure 3A. Other differences are also observed in the metabolic pathway enrichment, where PapMV→PRSV and PapMV are the only conditions that upregulate photosynthetic pathways, which are downregulated in PRSV. Also, PapMV→PRSV is the only condition where the plant–pathogen interaction pathway is upregulated (Figure 5). Antagonism and synergism share a common background as they are mixed infections of the same two viruses with only a few changes in gene expression. Likely, host cell availability leads to one or another type of interaction. Our transcriptome of synergism is similar to that reported in previous work with monocotyledons, PMV, and SPMV [27], as the single infection of PMV upregulates defense-related genes like *WRKY*, *PR*, and *PRR*. However, when PMV and SPMV are co-infected and synergism develops, the expression of defense-related genes is suppressed. Also, genes involved in responses to hormones and RNAi machinery are downregulated or non-differentially expressed.

Antagonism (PapMV→PRSV) is a complex interaction that seems to involve innate and adaptive immunity (Table 1), as transcripts from three out of the four different resistance/defense strategies against viruses [2] were differentially expressed: dominant resistance, RNAi-mediated resistance, and hormone-mediated resistance. Dominant resistance is present through the expression of *TIR-NBS-LRR* and *LRR-RK*. We hypothesize that this resistance would be primarily triggered by ethylene as we found the presence of several transcripts from genes such as *NPR1*, *ETO1*, *ERF109*, *ERF4*, and *ERF1A* (Table S3, Supplementary Materials), which are involved in the molecular signaling cascade of the plant–pathogen interaction pathway [8,9,54,55]. The response to ethylene also occurs in the single infections of PRSV and PapMV; however, in PapMV→PRSV (antagonism), it is especially enriched (Figure 3A and Figure S2, Supplementary Materials) with the contribution of each virus. The response to other hormones, such as salicylic acid (Figure 4), is also present in this condition but might not be enough for the defense response. The RNAi-mediated resistance is also involved here, as we found all the principal genes *AGO 2*, *DLC-2*, *DCL-4*, and *RDR* (Table S3, Supplementary Materials) overexpressed only in antagonism. Potyviruses can suppress the RNAi machinery through the induction of host *CML38* and *FRY1*, which negatively regulates silencing [56]. We found this also to be the case in PRSV infection, but not in PapMV→PRSV. This suggests that, in antagonism, the two types of resistance of the innate immune system (dominant and hormone-mediated) triggered by PRSV cannot overcome the three resistance mechanisms ignited by PapMV→PRSV in antagonism (dominant and hormone-mediated of the innate system), in addition to the RNAi of the adaptive immunity.

Thus, how does PapMV→PRSV infection leads to antagonism? We propose three possibilities, the first of which is a sort of cooperative activation of the plant immune system, such that PapMV initially slowly and smoothly triggers innate and adaptive immunity, and the addition of the innate system of PRSV (through the upregulation of a larger number of genes) is enough to reach antagonism. In the second alternative, PapMV quickly triggers the innate immune response only in the early steps of the infection, thereby interfering with the replication of PRSV. Finally, PapMV replication and movement in the host become much faster than PRSV [31], and it sets on a fraction of cells that PRSV can no longer use for replication, competing for available cells in a superinfection exclusion model [23,56,57,58,59]. Further studies are needed to deeply understand the antagonistic interaction between plant viruses. Dissecting the plant immune response is an opportunity to understand the development of resistance in important viral mixed crop diseases [60,61]. As PRSV is a damaging virus for many producers, the combination of immune responses from the plant and its manipulation would enable the creation of measures to counteract the reduction of production caused by this potyvirus.

## Figures and Tables

**Table 1:** Number of differentially expressed genes associated with immunity and resistance under each condition of single or mixed infections. PapMV—Papaya mosaic virus; PRSV—Papaya ringspot virus.

Condition	Innate Immunity			Adaptive Immunity
	Dominant resistance	Recessive resistance	Hormone mediated resistance	RNA interference mediated resistance
PapMV	● (12)	N/A *		● (3)
PRSV	●● (25)	N/A *	●● (15)	
PapMV→PRSV (Antagonism)	●●●● (76)	N/A *	●●● (33)	●● (15)
PapMV + PRSV (Synergism)	●● (16)	N/A *	● (5)	

● (*n*), the number of upregulated differentially expressed genes for each type of resistance (0 < ● < 15, 15 < ●● < 30, 30 < ●●● < 65, 65 < ●●●● < 100); * does not apply to this model as there are no reported mutations in the eukaryote translation initiation factors (eIFs) of the papaya gene family conferring resistance to these viruses. Also, genes involved in recessive resistance did not differentially express against the mock inoculated plants (immunity nomenclature from Nicaise [2]). The complete list of the genes involved is included in Table S3 (Supplementary Materials).

## Supplementary Materials

The following materials are available online at https://www.mdpi.com/1999-4915/12/2/230/s1: Figure S1. Dendrogram of sample clustering to detect outliers; Table S1. Quantitative expression, through qRT-PCR, of selected genes and their comparison to RNA-Seq data; Figure S2. Networks of the non-redundant biological process. (**A**) PapMV→PRSV, (**B**) PapMV + PRSV, (**C**) PapMV, and (**D**) PRSV. Edges represent relationships between the BPs. Node color intensity according to the *p*-value scale; Table S2. Statistics of RNA-Seq raw data and reads alignment to the de novo transcriptome; Table S3. Gene identity of innate and adaptive immunity for each treatment.
